# Impact of Cold Atmospheric Plasma (CAP) Treatments on the Oxidation of Pistachio Kernel Lipids

**DOI:** 10.3390/foods11030419

**Published:** 2022-01-31

**Authors:** Roberta Foligni, Cinzia Mannozzi, Lama Ismaiel, Filippo Capelli, Romolo Laurita, Silvia Tappi, Marco Dalla Rosa, Massimo Mozzon

**Affiliations:** 1 Department of Agricultural, Food and Environmental Sciences (D3A), Università Politecnica delle Marche, Via Brecce Bianche 10, 60131 Ancona, Italy; r.foligni@staff.univpm.it (R.F.); l.ismaiel@pm.univpm.it (L.I.); 2 Department of Industrial Engineering (DIN), University of Bologna, Via Terracini 24, 40131 Bologna, Italy; filippo.capelli@unibo.it (F.C.); romolo.laurita@unibo.it (R.L.); 3 AlmaPlasma s.r.l., Viale G. Fanin 48, 40127 Bologna, Italy; 4 Interdepartmental Centre for Industrial Research Health Sciences and Technologies, Alma Mater Studiorum-University of Bologna, Via Zamboni 33, 40136 Bologna, Italy; 5 Department of Agricultural and Food Sciences (DISTAL), University of Bologna, Piazza Goidanich 60, 47521 Cesena, Italy; silvia.tappi2@unibo.it (S.T.); marco.dallarosa@unibo.it (M.D.R.); 6 Interdepartmental Centre for Industrial Agrofood Research (CIRI Agrofood), University of Bologna, Via Quinto Bucci 336, 47521 Cesena, Italy

**Keywords:** cold atmospheric plasma (CAP), lipid oxidation, dry fruit, oxysterols, pistachio, *Pistacia vera*, volatiles

## Abstract

Cold atmospheric plasma (CAP) is a non-thermal technology that could be applied for food decontamination from both biological (microorganisms) and chemical (pesticides, food allergens, mycotoxins) contaminants, thanks to the production of reactive species (RS). However, RS could also promote the onset and the progress of food lipid oxidation, which may limit the quality and acceptability of the final products. The aim of this work was to assess the oxidation degree of pistachio kernels after treatment in a surface dielectric barrier discharge (SDBD). Two different operative conditions for CAP generation were investigated, resulting in the production of high (800 ppm) or low (300 ppm) concentrations of ozone. Limited amounts of hydroperoxides (3.00–4.22 mEq O_2_/kg), thiobarbituric acid reactive substances (TBARS, 0.072–0.600 mg TEP/g oil), and phytosterol oxidation products (POPs, 14.43–17.20 μg/g) were observed in lipids of both control and plasma processed pistachios. Plasma treatments did not significantly affect the total fatty acid composition and the amounts of identified unsaponifiable matter constituents (4-desmethylsterols, 4,4-dimethylsterols, 4-methylsterols), except for an unexpected significant increase of γ-tocopherol content in extracted oils. These findings contribute to gaining further knowledge for the scale-up of CAP technology to industrial processing.

## 1. Introduction

The pistachio tree (*Pistacia vera* L.) produces seeds whose kernels are eaten whole (fresh or roasted and salted) and used as ingredient in a wide variety of traditional foods in several countries, such as mortadella, gelato, and spumone (Italy), baklava (Iran, Turkey), kulfi (Indian subcontinent), or lokum (Turkey). The worldwide production of pistachio nuts was around 1 million t/year in 2015–2019, with the three major producers (Iran, Turkey, USA) accounting for about 85% of the total production [1]. Despite the limited contribution to the global production of pistachios (less than 0.5%), Italy has a significant history in the cultivation and processing of pistachio nuts: in 2010 the Green Pistachio coming from the small Sicilian town of Bronte was awarded the PDO (Protected Designation of Origin) denomination [2].

The contamination by aflatoxin-producing fungi represents a serious threat to the safety of this valuable commodity, especially during the postharvest storage of nuts, eventually causing economic losses to the producing countries. Different strategies (physical, chemical, and biological) have been suggested to overcome the problem [3,4], but these treatments have been proved to be not completely suitable for different reasons (reliability, sensorial acceptability of the final product, production of toxic residues, costs). This has driven the researchers’ efforts to the development of new methods to prevent mold growth and to destroy mycotoxins.

Cold atmospheric plasma (CAP) technologies have been recently assessed for their ability to control fungal growth in several kinds of nuts (hazelnuts, peanuts, pistachio), thus reducing mycotoxin formation [5,6]. Better known as the fourth state of matter, plasma is partially ionized gas made up of radicals, ions, electrons, reactive neutral species and/or excited particles, and UV photons. A huge potential of CAP consists in the low temperature reached during the treatment, which results close to room temperature (<35 °C) due to the local non-equilibrium conditions between electrons and heavier particles [7,8]. Both atmospheric and low-pressure plasma generating devices have been assessed for their effectiveness on pistachios: 1–5 log reductions of *Aspergillus parasitucus* [9], 2–5 log reductions of *A. flavus* [10,11,12,13], and 1–2 log reductions in the fungal population of naturally contaminated pistachios (range: 5.67–7.96 log CFU g^−1^) [14] have been measured, according to the device design, type of feeding gas (air, argon, sulfur hexafluoride), and exposure time. More recently, the effect of a plasma jet source on the warehouse pest of pistachio *Plodia interpunctella* has been evaluated as an alternative method of non-toxic pest control [15]. Even if the chemistry of the process is currently under investigation, it is a fact that plasma treatments showed strong potentials in mycotoxins degradation as well: [9] observed 20–50% reduction in the aflatoxin levels (B1, B2, G1, and G2), while [10] reported a 52% reduction of B1 toxin in pistachio nut samples.

Reactive oxygen species (ROS), reactive nitrogen species (RNS), charged particles, excited molecules, and UV photons have been suggested to be involved in the mechanisms of microbial inactivation [16,17]. On the other side, plasma reactive species could trigger and/or increase the occurrence of lipid rancidity, especially in food matrices sensitive to this spoilage due to their high lipid content and/or unsaturation degree and/or low water activity. Despite the number of studies on the decontamination effects of cold plasma on pistachio nuts, only very few of them investigated the potential consequences of plasma processing on kernel lipid integrity. Makari et al. [10] reported that the exposure time of pistachio kernels in a dielectric barrier discharge (DBD) air plasma system significantly affected the content of thiobarbituric acid reactive substances (TBARS). Other physicochemical changes originated from oxidative events were observed by the same authors: plasma processed pistachios showed lower levels of chlorophylls and carotenoids and lower proteins solubility than raw nuts. Plasma treatment did not significantly affect the sensory attributes of nuts (odor, appearance, texture), thus supposing that if some oxidation occurred, its consequences were not perceivable [9,12,14]. However, Makari et al. [10] observed instrumental color (L, a*, b*, C*) changes during plasma treatment that resulted in darker pistachio nuts color at longer plasma exposure time.

For the reasons stated above, we intended to provide more detailed information on the oxidative state of pistachio kernel lipids after treating the nuts in a CAP system equipped with a surface dielectric barrier discharge (SDBD). Global indexes (peroxide value, TBARS amount) and direct analytical determinations of both volatile (short-chain alcohols, aldehydes, and ketones) and non-volatile (phytosterols oxidation products, POPs) substances were carried out to provide a whole assessment of the oxidative conditions of pistachio kernel oil.

## 2. Materials and Methods

### 2.1. Sampling and Plasma Treatments

Pistachio (*Pistacia vera* L.) nuts were purchased from a local distributor in Italy. The samples were peeled and mixed, considering the distribution of lipid might have differed throughout the samples in accordance with the geographic origin of pistachio kernels [18]. Nuts were split into portions of 23 ± 2 g each and submitted to plasma treatment for 30 min at a distance of 16.5 cm from the plasma source and to further analysis. All experiments were conducted in triplicate.

An SDBD plasma source operated in ambient air [8] was used for sample treatments. Two different operative conditions were investigated, both working with sinusoidal high voltage signal having a peak voltage of 6.6 kV and a frequency of 23 kHz, varying the duty-cycle (DC), i.e., the ratio of time in which the signal was turned on over the treatment time. The ozone concentrations were measured using the optical absorption spectroscopy technique that exploits the Lambert–Beer law [8], resulting in low (300 ppm, O_3_) or high (800 ppm, O_3_^(+)^) ozone concentrations using 10% DC or 100%DC, respectively. The untreated sample was kept as a control.

### 2.2. Total Lipid Extraction and Lipid Analyses

Total lipid extraction was performed at room temperature according to Bligh and Dyer [19]. Briefly, each portion (23 ± 2 g) of pistachio kernels was homogenized with a proper volume of chloroform/methanol mixture 1:1 *v*/*v*, in such proportions that a monophasic system was formed with the water in the sample (chloroform/methanol/water ratio 1:1:0.4 *v*/*v*/*v*). Following dilution with aqueous potassium chloride 0.88% (chloroform/methanol/water ratio 1:1:0.9 *v*/*v*/*v*) separated the solvent mixture into 2 layers, the upper one (chloroform layer) containing the lipids. The solvent was removed in a rotary evaporator, and kernel oils were stored at −20 °C until analyses.

Peroxide value (PV) was determined according to AOCS Official Method Cd 8b-90 [20] and expressed as mEq O_2_/kg oil.

The quantitative determination of TBARS amount in pistachio oil samples was carried out according to the procedure described by Pegg [21]. Results were expressed as milligrams of TEP (1,1,3,3-tetraethoxypropane)/ g oil.

Fatty acids methyl esters (FAMEs) were prepared from lipid extracts by acid-catalyzed transesterification, according to Tavoletti et al. [22], and analyzed in a Trace 1300 gas chromatograph (Thermo Fisher Scientific, Waltham, MA, USA) equipped with a flame ionization detector (FID) and a TG-Polar capillary column 60 m × 0.25 mm i.d., 0.20 μm film thickness (Thermo Fisher Scientific, Waltham, MA, USA) in the operative conditions described in [23]. A standard mixture of 37 FAMEs (Supelco-Bellefonte, Pennsylvania, PA, USA) was used for the identification of chromatographic peaks. The total fatty acid (FA) compositions (weight % of total FA) were calculated by the peak area normalization method.

The unsaponifiable matter was prepared by overnight cold saponification as described by [24], after the addition of 1 mL of 5α-cholestane (1000 ppm in hexane) and 25 µL of 19-hydroxycholesterol (500 ppm hexane/isopropanol 3:2 *v*/*v*) to about 250 mg (exactly weighted) of the sample, as internal standards for total sterols and POPs quantification, respectively. POPs were concentrated by SPE (solid phase extraction) silica cartridges (500 mg, 6 mL; Supelco-Bellefonte, PA, USA) preconditioned with 10 mL of hexane. After sample deposition, the cartridge was eluted with 2 mL of hexane/diethyl ether mixture 75:25 *v*/*v*, 6 mL of hexane/diethyl ether 60:40 *v*/*v*, and 4 mL of acetone (POPs collection). Total unsaponifiable matter and POPs fraction were derivatized for gas chromatography (GC) analysis by a ready-to-use silylating mixture (pyridine/chlorotrimethylsilane/hexamethyldisilazane 10:1:2 *v*/*v*/*v*; Supelco-Bellefonte, PA, USA). A Trace 1300 gas chromatograph coupled with a ISQ 7000 single quadrupole mass spectrometer (Thermo Fisher Scientific, Waltham, MA, USA) and equipped with a Zebron ZB-5 capillary column 30 m × 0.25 mm i.d., 0.25 μm film thickness (Phenomenex, Torrance, CA, USA) were used for the gas chromatography—mass spectrometry (GC-MS) analysis of sterols and their oxidation products. A Dual Detector Microfluidics kit (Thermo Fisher Scientific, Waltham, MA, USA) was used to split 1:1 the injected sample between the MS and the GC-FID. The kit allows keeping the split ratio constant by working in constant pressure mode (Helium 110 kPa). Injector (splitless mode), FID, and transfer line were at 300 °C, while the ion source was set at 320 °C. The oven temperature increased from 90 to 290 °C at the rate of 30 °C/min, then increased to 300 °C at the rate of 1 °C/min and was kept at this value for 15 min. Electronic impact (EI) fragmentation was collected in the range 50–650 a.m.u. for peak identification, while the FID signal was used for the quantification of the unsaponifiable matter components, according to the internal standard method.

### 2.3. Analysis of Volatile Components

Volatiles were collected from both kernel oils and ground kernels by headspace solid-phase microextraction (HS-SPME) and analyzed by the GC-MS instrument previously described. The operative parameters for volatiles sampling and GC-MS analysis were summarized in Belleggia et al. [25].

Volatile compounds were identified by matching mass spectral data, and Kovats Retention Indices (RIs) with those collected in the NIST/EPA/NIH Mass Spectral Library 2020 A C8–C20 normal alkanes mixture (Sigma-Aldrich, St. Louis, MO, USA) was used to calculate RIs in the experimental conditions. An automated spreadsheet was used to simplify the calculation of RIs of the unknown components [26].

### 2.4. Data Analysis

The Tukey–Kramer’s honest significant difference (HSD) test was used to compare the experimental variables between treatments (O_3_, (i.e., samples treated using 10% DC with an ozone level of 300 ppm), O_3_^(+)^, (i.e., samples treated using 100% DC, with an ozone level of 800 ppm) and (C) control). All statistical analyses were carried out by the software JMP^®^ Version 10 (SAS Institute Inc., Cary, NC, USA).

## 3. Results and Discussion

Table 1 summarizes the analytical data concerning the characterization of the samples (total FA and phytosterol compositions) and the quantification of primary (hydroperoxides) and secondary (TBARS, POPs) oxidation products of pistachio kernel lipids.

Three FAs (palmitic, C16:0; oleic C18:1Δ9; linoleic, C18:2Δ9,12) accounted for 95% of total FAs. The low oleic/linoleic ratio did not come out in favor of an Italian origin of the nuts, as suggested by Arena et al. [18] and by comparison with data collected from pistachios of different origins [27,28,29].

Five 4-desmethylsterols (cholesterol, campesterol, stigmasterol, β-sitosterol, and Δ^5^-avenasterol) were identified in the unsaponifiable matter of pistachio oils by MS. The cleavages of C1-C10 and C3-C4 bonds in the A ring gave intense peaks at *m*/*z* 129 and [M − 129]^+^, which characterized the EI mass spectra of Δ^5^-sterols. Two 4,4-dimethylsterols (triterpenic alcohols, triterpenols) were detected as well, whose fragmentation patterns were consistent with cycloartenol and 24-methylenecycloartanol. Their mass spectra showed abundant correlated ions due to loss of trimethylsilylhydroxy [TMSOH] and methyl groups, which appeared at *m*/*z* 408 and 393 for cycloartenol, and at *m*/*z* 422 and 407, for 24-methylenecycloartanol. Typically, 9,19-cyclopropanesterols contain a triplet of fragment ions at [M − 133]^+^, [M − 159]^+^, and [M − 212]^+^, the latest due to cleavages of C9-C19, C9-C10, and C5-C6 bonds (ring B and cyclopropane): these ions were observed at *m*/*z* 365, 339, 286, and 379, 353, 300, for cycloartenol and 24-methylenecycloartanol, respectively. Citrostadienol was the only 4-methylsterol identified in the chromatographic conditions adopted. The base peak at *m*/*z* 357 was attributed to the most frequently represented fragment in Δ^7^-sterols with an unsaturated side chain (SC) and corresponding to [M − SC − 2]^+^: side chain was thus determined to be C_10_H_19_. The presence of the correlated ions [M − SC − TMSOH]^+^, [M − SC − TMSOH − 2]^+^, and [M − SC − C_3_H_5_ − TMSOH]^+^ (*m*/*z* 269, 267, and 227, respectively) confirmed this conclusion. Tocols were easily distinguishable by means of MS, as their molecular ion and fragmentation showed the type of isomer (α, β, γ, δ) and the type of side chain (saturated; mono, di, or tri unsaturated): the three most abundant peaks correspond to molecular ion [M]^+^, an ion derived from the loss of the side chain, and an ion originated from the cleavage of the side chain accomplished by the loss of methylacetylene fragment. Based on MS data, we identified the 7,8-dimethyl (γ isomer) tocopherol [30,31,32].

The total content of 4-desmethylsterols is consistent with data found by Wang et al. [31] in pistachio nuts coming from Chinese markets and by Lucarini et al. [33] in raw pistachio kernels coming from the PDO Bronte’s area (Sicily, Italy). Lower values of sterol amount in pistachio oils were reported by Yahyavi et al. [27] in samples collected from different parts of East Azarbaijan (Iran). β-sitosterol was the dominant 4-desmethylsterol, accounting for 85.6–86.5% of total 4-desmethylsterols, followed by Δ5-avenasterol, campesterol, and stigmasterol, agreeing with previously published data [18,27,33]. The content of cycloartenol in kernel oils (8.4–8.9 mg/100 g oil) was higher than levels observed by Wang et al. (2.9 ± 0.7 mg/100 g oil)) [31], while 24-methylenecycloartanol levels (9.5–11.1 mg/100 g oil) were lower than values reported by the same authors (21.8 ± 6.4 mg/100 g). γ-tocopherol has been reported to be the most abundant tocol in *Pistacia vera* kernel oil, accounting for 85–93% of total tocols [27]. The γ-tocopherol amount of control and processed nuts (32.3–50.0 mg/100 g oil) was higher than values reviewed by Catalán et al. (8.0–27.4 mg/100 g) [29] in pistachio oils of different origins and values reported by Yahyavi et al. [27] (11.2–23.2 mg/100 g oil) in pistachios grown in East Azarbaijan (Iran) but agreed with γ-tocopherol contents found in *Pistacia atlantica* Desf. oils (42.0–44.6 mg/100 g) [28].

Both plasma treatments did not significantly affect the FAMEs composition and the amounts of identified unsaponifiable matter constituents (4-desmethylsterols, 4,4-dimethylsterols, and 4-methylsterols). An unexpected significant increase of γ-tocopherol was observed in plasma-treated kernels, especially in O_3_(+) samples. Makari et al. [10] observed a little increase of antioxidant activity in some plasma-treated samples of pistachio nuts and hypothesized that it could be due to cell membrane breakdown caused by reactive plasma species, thus promoting the release of membrane-bound antioxidants.

Global indexes (PV, TBARS) showed a limited oxidation degree of samples, scarcely affected by the plasma treatment and mostly ascribable to preexisting oxidative spoilage of nuts. On the contrary, Ref. [10] reported that the exposure time of pistachio kernels in a DBD plasma generating device significantly affected the TBARS amount. Measured PVs (3.00–4.22 mEq O_2_/kg oil) were consistent with the values reported by [28] for *Pistacia atlantica* Desf. oils extracted with different methods (1.91–2.15 mEq O_2_/kg) and by [18] in Italian pistachio oils (2.9–6.8 mEq O_2_/kg), while [27] observed lower values (0.19–1.0 mEq O_2_/kg) in Iranian oils.

**Figure 1:**
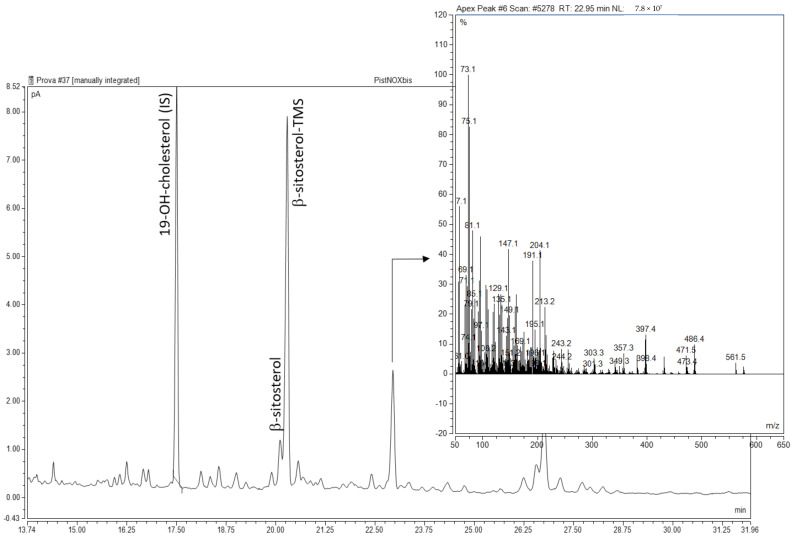
GC-FID chromatogram of the SPE polar fraction of the unsaponifiable matter of pistachio kernel oil and mass spectrum of the hypothesized POPs peak.

As FA hydrocarbon chains, phytosterols could also undergo the well-known free-radical oxidation pathway, leading to primary oxidation products (hydroperoxides, epoxydes), which decomposed to secondary oxidation products with different functional groups (hydroxy and keto). In the chromatographic conditions adopted for the analysis of the polar fraction of the unsaponifiable matter, only one peak showed a mass fragmentation pattern related to trimethylsilyl (TMS) derivatives of sterols. Ref. [33] identified one keto and three hydroxy derivatives of sitosterol in raw pistachio kernels (Figure 1). However, the mass spectra of the unknown peak did not clearly match the fragmentation patterns of the well-characterized POPs [34,35], such as 7-hydroxy, 5,6-epoxy, 7-keto, and triol derivatives of the phytosterols identified in pistachio oil samples. As suggested by [36], some overlapping might occur between POPs analyzed on DB-5 type capillary columns. Ions at *m*/*z* 486 [M]^+^, 471 [M − CH_3_]^+^, and 396 [M − TMSOH]^+^ were consistent with 7-ketocampesterol. The characteristic fragment ion at *m*/*z* 431 [M − TMSOH − 71]^+^ could originate from the di-TMS derivative of sitostanetriol. Indeed, the same kind of fragment characterized the mass spectra of pure cholestane-3,5,6-triol (Merck KGaA, Darmstadt, Germany), while the molecular ions corresponding to both di- and tri-TMS derivatives were not visible. The quantification of the POPs peak by the internal standard method gave a total POPs content of 14.43–17.20 μg/g oil, which was unaffected by plasma treatments. [33] reported similar values of total POPs content in raw pistachio kernels (4.40 ± 0.14 μg/g oil).

**Figure 2:**
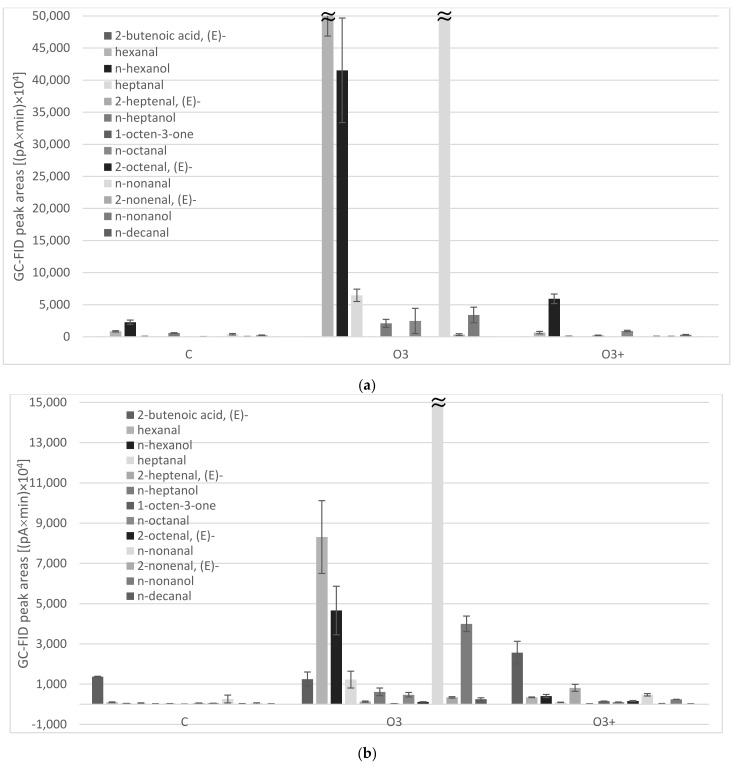
Volatile oxidation markers abundances as sampled by headspace solid-phase microextraction and analyzed by GC-FID/MS: (**a**) ground pistachio kernels; (**b**) pistachio kernel oils obtained by cold solvent extraction. C = control (untreated samples), O_3_ = samples treated using 10% DC (ozone level 300 ppm); O_3_^(+)^ = samples treated using 100% DC (ozone level 800 ppm).

TBARS data were consistent with the results of the analyses of volatile substances (Table 2). A total of 29 volatiles were identified in the headspace of ground kernels and their lipid fractions, as sampled by a grey (Divinylbenzene/Carboxen/Polydimethylsiloxane) SPME fiber. Monoterpenes (β-thujene, α-pinene, 3-carene, p-cymene, limonene, β-ocimene, terpinolene) dominated in the headspace of control (C) and high-power processed kernels (O_3_^(+)^), while a strong increase in key markers of lipid peroxidation (C6–C10 aldehydes, alcohols, and ketones) was observed in pistachios treated using 10% DC (O_3_). Particularly, nonanal and a couple of strictly related substances hexanal/hexanol were the most abundant ones (Figure 2a). As reported by Brewer [37], they originated from beta-cleavage of hydroperoxide isomers of oleic and linoleic acid, respectively, which were the two most represented FAs in pistachio samples, accounting for about 85% of total FAs. This behavior was even more clear in kernel oil samples, where volatile oxidation markers always predominated in the headspace of pistachio oils. The impact of plasma treatment on lipid oxidation was evident in O_3_ samples, mainly due to the production of hexanal, hexanol, and nonanal but other oxidation markers originated by oleic and linoleic acids (heptanol, octanal, nonanol) significantly increased (Figure 2b).

On the other side, other compounds (such as 2-pentanone, 4-hydroxy-4-methyl, α-pinene, 2-heptenal, (E)-, 1-octen-3-one, methyl 5-oxohexanoate, 2-octenal, (E)- and n-tridecane) were found to be significantly higher in the kernel oil of O_3_^(+)^ sample. Since this sample was subjected to a higher ozone concentration, it is expected to show a more advanced oxidation status compared to the O_3_ one. The volatile compounds content did not show a clear trend. This might be due to the complexity of the oxidation process and to the possible formation of tertiary products that were not detected in the present research.

Despite the clear oxidation signs in the plasma treated samples, no appreciable changes in odor, texture, and overall appearance of pistachio kernels were observed, according to the reports of several researchers [9,12,14].

## 4. Conclusions

Atmospheric pressure devices can generate cold plasma in the surrounding air, thus simplifying the treatment chamber design and the scale-up to industrial food processing. However, plasma reactive species can promote the onset and progress of oxidative degradation of food lipids, and pistachio nuts are quite susceptible to that due to the FA composition and low water activity of the matrix. The efficiency of CAP in nuts decontamination from fungi and their toxic metabolites (mycotoxins) should hence be assessed in view of its potentially negative consequences on sensory and nutritional properties.

Even if non-volatile (hydroperoxides, TBARS, POPs) and volatile (C6-C10 aldehydes and alcohols) key markers of lipid peroxidation were observed in treated pistachio kernels, and their relationship with ozone concentration during treatment was not clear, their amount was not significantly different between treated and control samples and did not cause significant changes in the whole composition of kernel lipids ((fatty acids, alcoholic constituents of unsaponifiable matter). These results come out in favor of the safety of cold plasma treatments at atmospheric pressure, which is strictly necessary for regulatory approval of plasma technologies from governmental bodies.

Further studies are needed to better understand the relationships between lipid oxidation and the operative variables (power density, exposure time), also in relation to the efficiency of microbial and toxin decontamination.

## Figures and Tables

**Table 1:** Analytical data of pistachio kernel lipids (mean ± SD, n = 3) treated by an SDBD plasma generating device.

Parameter	C ^1^	O_3_	O_3_^(+)^
Peroxide value [mEq O_2_/kg oil]	3.36 ± 0.69	3.00 ± 0.42	4.22 ± 0.78
TBARS [mg TEP/g oil]	0.072 ± 0.004 ^b^	0.600 ± 0.037 ^a^	0.109 ± 0.007 ^b^
Total FAMEs composition [%] ^2^			
C14:0	0.13 ± 0.06	0.19 ± 0.05	0.10 ± 0.05
C16:0	9.68 ± 0.51	11.17 ± 0.74	9.86 ± 0.59
C16:1	0.73 ± 0.07 ^b^	1.07 ± 0.14 ^a^	0.90 ± 0.14 ^ab^
C17:0	0.03 ± 0.01	0.37 ± 0.54	0.05 ± 0.02
C18:1Δ9	52.21 ± 1.50	50.70 ± 1.52	50.03 ± 1.99
C18:1Δ11	2.03 ± 0.18	2.08 ± 0.19	1.68 ± 0.17
C18:2Δ9,12	34.07 ± 1.88	33.19 ± 2.64	36.66 ± 0.73
C18:3Δ9,12,15	0.52 ± 0.05	0.47 ± 0.07	0.61 ± 0.07
C20:0	0.10 ± 0.03	0.14 ± 0.06	0.06 ± 0.03
C20:1Δ11	0.38 ± 0.01	0.46 ± 0.13	0.37 ± 0.01
C21:0	0.07 ± 0.02	0.12 ± 0.03	0.10 ± 0.03
C20:2Δ11,14	0.03 ± 0.00	0.04 ± 0.01	0.03 ± 0.01
Unsaponifiable matter components			
cholesterol [mg/100 g oil]	6.1 ± 0.0 ^a^ (2.0 ± 0.3)	3.2 ± 0.4 ^b^ (1.1 ± 0.2)	5.6 ± 0.2 ^a^ (1.8 ± 0.3)
campesterol [mg/100 g oil]	14.1 ± 1.5 (4.5 ± 0.1) ^3^	15.7 ± 2.2 (5.2 ± 0.6)	14.3 ± 1.9 (4.6 ± 0.2)
stigmasterol [mg/100 g oil]	3.0 ± 0.6 (1.0 ± 0.1)	3.6 ± 0.2 (1.2 ± 0.1)	3.2 ± 0.6 (1.0 ± 0.0)
β-sitosterol [mg/100 g oil]	266.0 ± 33.6 (85.8 ± 0.1)	259.5 ± 18.8 (85.6 ± 0.8)	269.1 ± 50.3 (86.5 ± 0.9)
Δ^5^-avenasterol [mg/100 g oil]	20.9 ± 4.0 (6.7 ± 0.4)	20.8 ± 1.6 (6.9 ± 0.8)	18.8 ± 2.0 (6.1 ± 0.4)
Total 4-desmethylsterols [mg/100 g oil]	310.2 ± 39.7	302.9 ± 19.3	310.9 ± 55.1
cycloartenol [mg/100 g oil]	8.9 ± 0.2	8.4 ± 0.8	8.6 ± 1.9
24-methylenecycloartanol [mg/100 g oil]	9.5 ± 1.3	11.1 ± 0.7	9.6 ± 2.2
Total triterpenols [mg/100 g oil]	18.4 ± 1.1	19.5 ± 1.4	18.1 ± 4.1
citrostadienol [mg/100 g oil]	5.9 ± 1.4	6.1 ± 1.1	7.9 ± 2.4
γ-tocopherol [mg/100 g oil]	32.3 ± 3.0 ^b^	35.6 ± 7.9 ^ab^	50.0 ± 6.8 ^a^
POPs [μg/g oil]	14.47 ± 3.72	14.43 ± 5.32	17.20 ± 5.54

^1^ Column heads are: C = control (untreated samples), O_3_ = samples treated using 10% DC (ozone level 300 ppm); O_3_^(+)^ = samples treated using 100% DC (ozone level 800 ppm). Values in a row with different letters are significantly different (Tukey test, *p* < 0.05). ^2^ Cm:n Δx, m = number of carbon atoms; n, number of double bonds; x, position of double bonds. ^3^ Percentage of total 4-desmethylsterols.

**Table 2:** Volatile compounds (mean ± SD; *n* = 3) ^1^ detected in the headspace of pistachio kernel oils and ground kernels treated by an SDBD plasma generating device.

RI ^3^		Kernel Oils			Ground Kernels		
		C ^2^	O_3_	O_3_^(+)^	C	O_3_	O_3_^(+)^
794	2-butenoic acid, (E)-	1374 ± 18	1250 ± 354	2566 ± 564			
799	hexanal	101 ± 18 ^b^	8310 ± 1808 ^a^	347 ± 17 ^b^	849 ± 101 ^b^	54,606 ± 7741 ^a^	651 ± 187 ^b^
841	2-pentanone, 4-hydroxy-4-methyl-	128 ± 17 ^b^	113 ± 14 ^b^	234 ± 18 ^a^			
870	*n*-hexanol	33 ± 12 ^b^	4659 ± 1206 ^a^	410 ± 78 ^b^	2274 ± 332 ^b^	41,530 ± 8147 ^a^	5922 ± 740 ^b^
902	heptanal	48 ± 23 ^b^	1226 ± 417 ^a^	95 ± 9 ^b^	75 ± 13 ^b^	6463 ± 964 ^a^	105 ± 3 ^b^
909	butyrolactone	206 ± 17	296 ± 66	387 ± 59			
930	β-thujene				265 ± 7 ^b^	620 ± 30 ^a^	657 ± 7 ^a^
939	α-pinene	996 ± 18 ^b^	1036 ± 50 ^b^	1615 ± 221 ^a^	4390 ± 304 ^b^	4943 ± 277 ^b^	17742 ± 322 ^a^
960	2-heptenal, (E)-	28 ± 5 ^b^	133 ± 30 ^b^	817 ± 174 ^a^			
967	benzaldehyde	147 ± 15	77 ± 26	78 ± 16			
972	*n*-heptanol	36 ± 2 ^b^	618 ± 190 ^a^	26 ± 8 ^b^	585 ± 57 ^ab^	2094 ± 623 ^a^	198 ± 65 ^b^
979	1-octen-3-one	19 ± 1 ^c^	33 ± 3 ^b^	158 ± 3 ^a^			
995	3,5-dimethyl-2(5H)-furanone	214 ± 123	143 ± 5	276 ± 18			
1000	*n*-decane	97 ± 69	22 ± 5	111 ± 8			
1003	*n*-octanal	56 ± 9 ^c^	475 ± 112 ^a^	106 ± 8 ^b^	31 ± 7 ^c^	2473 ± 1962 ^a^	902 ± 112 ^b^
1012	3-carene				242 ± 22	413 ± 147	403 ± 30
1020	methyl 5-oxohexanoate	223 ± 31 ^b^	256 ± 49 ^ab^	438 ± 67 ^a^			
1025	*p*-cymene				898 ± 122 ^a^	843 ± 96 ^a^	301 ± 7 ^b^
1035	limonene	629 ± 62	943 ± 170	806 ± 158	7058 ± 698 ^ab^	10,867 ± 1545 ^a^	5491 ± 266 ^b^
1038	β-ocimene				254 ± 37^b^	598 ± 98^a^	302 ± 19^b^
1062	2-octenal, (E)-	49 ± 5 ^b^	119 ± 11 ^ab^	160 ± 27 ^a^			
1080	terpinolene				468 ± 75 ^b^	2076 ± 440 ^a^	872 ± 8 ^b^
1100	*n*-undecane	271 ± 32	304 ± 64	524 ± 93	1507 ± 308	992 ± 204	1511 ± 23
1105	*n*-nonanal	263 ± 193 ^b^	36,123 ± 9035 ^a^	472 ± 60 ^b^	395 ± 112 ^b^	72,676 ± 20,053 ^a^	90 ± 4 ^b^
1163	2-nonenal, (E)-	28 ± 8 ^b^	344 ± 35 ^a^	31 ± 8 ^b^	51 ± 13 ^b^	337 ± 140 ^a^	64 ± 17 ^b^
1174	*n*-nonanol	40 ± 33 ^b^	3998 ± 385 ^a^	246 ± 1 ^b^	243 ± 38 ^b^	3402 ± 1213 ^a^	315 ± 49 ^b^
1200	*n*-dodecane	292 ± 70	484 ± 84	545 ± 93	1065 ± 257	1135 ± 301	868 ± 37
1207	*n*-decanal	22 ± 8 ^b^	253 ± 69 ^a^	26 ± 5 ^b^			
1300	*n*-tridecane	223 ± 2 ^b^	305 ± 35 ^ab^	335 ± 16 ^a^	362 ± 109	418 ± 139	197 ± 18

^1^ GC-FID peak areas [(pA × min) × 10^4^]. ^2^ Column heads are: C = control (untreated samples), O_3_ = samples treated using 10% DC (ozone level 300 ppm); O_3_^(+)^ = samples treated using 100% DC (ozone level 800 ppm). Values in a row with different letters are significantly different (Tukey test, *p* < 0.05). ^3^ Kovats Retention Index.

## Data Availability

The data presented in this study are available on request from the corresponding author. The data are not publicly available due to Intellectual Property protection.
